# Heavy metals contaminating the environment of a progressive supranuclear palsy cluster induce tau accumulation and cell death in cultured neurons

**DOI:** 10.1038/s41598-019-56930-w

**Published:** 2020-01-17

**Authors:** Carolina Alquezar, Jessica B. Felix, Elizabeth McCandlish, Brian T. Buckley, Dominique Caparros-Lefebvre, Celeste M. Karch, Lawrence I. Golbe, Aimee W. Kao

**Affiliations:** 1 Memory and Aging Center, Department of Neurology, University of California, San Francisco, California 94158 USA; 2 Graduate Program, Department of Molecular and Cellular Biology Baylor College of Medicine, Houston, Texas 77030 USA; 3 Environmental and Occupational Health Sciences Institute (EOHSI), Rutgers University, 170, Frelinghuysen Road Piscataway NJ, 08854 New Brunswick, NJ United States; 4 Centre Hospitalier de Wattrelos, 30 Rue Alexander Fleming, 59393 Wattrelos, cedex France; 5 Department of Psychiatry, Washington University in St Louis, St Louis, MO 63110 USA; 6 Division of Movement Disorders. Rutgers Robert Wood Johnson Medical School, New Brunswick, NJ United States

**Keywords:** Molecular medicine, Neurodegeneration

## Abstract

Progressive supranuclear palsy (PSP) is a neurodegenerative disorder characterized by the presence of intracellular aggregates of tau protein and neuronal loss leading to cognitive and motor impairment. Occurrence is mostly sporadic, but rare family clusters have been described. Although the etiopathology of PSP is unknown, mutations in the *MAPT*/tau gene and exposure to environmental toxins can increase the risk of PSP. Here, we used cell models to investigate the potential neurotoxic effects of heavy metals enriched in a highly industrialized region in France with a cluster of sporadic PSP cases. We found that iPSC-derived iNeurons from a *MAPT* mutation carrier tend to be more sensitive to cell death induced by chromium (Cr) and nickel (Ni) exposure than an isogenic control line. We hypothesize that genetic variations may predispose to neurodegeneration induced by those heavy metals. Furthermore, using an SH-SY5Y neuroblastoma cell line, we showed that both heavy metals induce cell death by an apoptotic mechanism. Interestingly, Cr and Ni treatments increased total and phosphorylated tau levels in both cell types, implicating Cr and Ni exposure in tau pathology. Overall, this study suggests that chromium and nickel could contribute to the pathophysiology of tauopathies such as PSP by promoting tau accumulation and neuronal cell death.

## Introduction

Progressive supranuclear palsy (PSP) is a relentless progressive neurodegenerative disorder that typically presents with postural instability, including gait and balance and the development of additional motor, cognitive and behavioral symptoms with the progression of the disease^1^. From a clinical standpoint, PSP is often classified among the parkinsonian disorders because of the bradykinesia and cogwheel rigidity due to the involvement of dopaminergic neurons in the brainstem and basal ganglia^2^. Neuropathologically, PSP is considered a tauopathy characterized by the presence of cytosolic aggregates of tau protein in affected neurons^3^. In tauopathies, pathological tau loses its affinity to bind to microtubules leading to the disruption of axonal transport and potentially contributing to neuronal death^4^. In contrast to other tauopathies such as Alzheimer’s disease (AD), PSP has relative low incidence (1,000 per 100,000 and 1–2 per 100,000 per year respectively)^5^.

The mechanisms underlying tau pathology and neuronal death in PSP are largely unknown. Although mutations in several genes, including *MAPT* (the gene encoding the tau protein) have been associated with PSP, most cases of the disease have no present genetic variations and/or mutations^6–10^. It has also been reported that the exposure to environmental toxins increases the risk of sporadic PSP^11–14^. Recently, a cluster of 92 sporadic PSP patients was documented in Wattrelos^15^, a small town in northern France home to metal-related industries since the mid-19^th^ century. The PSP patients in the Wattrelos cluster were not family related, had diverse genetic backgrounds and no known family history of PSP^15^. Therefore, although molecular genetic analysis had not been performed, the Wattrelos cluster seemed unlikely to be due to hereditary or genetic causes. Rather, the authors speculated that the presence of the PSP cluster could be related to environmental exposure to those heavy metals from improper disposal of industrial waste in residential areas^15^.

Heavy metals are metals that can have adverse effects on living organisms with a density higher than 5 g/cm^3^ ^16–18^. In humans, long-term exposure to elevated concentration of heavy metals is linked to several neurological disorders, including multiple sclerosis, Parkinson’s disease, Alzheimer’s disease and muscular dystrophy^19^.

From a toxicological perspective, understanding neuronal tolerance against heavy metal-induced stress could shed light on the causes of sporadic PSP and other neurodegenerative diseases. A recent report from the French government showed that the heavy metals chromium (Cr), nickel (Ni) and cadmium (Cd) were highly contaminating the environment in Wattrelos. Thus, we speculated that exposure to Cr, Ni and Cd could contribute to the development of PSP in the region of Wattrelos, France. We investigated the neurotoxic effects of chromium, nickel and cadmium using two different human cell models: induced pluripotent stem cell (iPSC)-derived neurons (iNeurons) carrying a PSP-related mutation in *MAPT* gene matched with a gene-corrected isogenic control line; and SH-SY5Y neuroblastoma cells (undifferentiated and neuron-like retinoic acid (RA)-differentiated). Our results showed that treatment with the three heavy metals induced cell death in a dose-dependent manner in iPSC-derived iNeurons. iNeurons carrying the R406W *MAPT*/tau mutation trended to be more sensitive to cell death induced by Cr and Ni treatments, however Cd exposure was equally toxic for both control and *MAPT* mutant cell lines. Furthermore, Cr and Ni exposure induced apoptotic cell death in SH-SY5Y cells, a well characterized dopaminergic neuronal-like cell model. Importantly, Cr and Ni treatments increased tau protein levels and phosphorylation in both SH-SY5Y cells and iNeurons. Together, the results presented here could link the neurotoxicity induced by these heavy metals with tau accumulation and pathology. Future work could investigate whether exposure to chromium and nickel directly contributes to the presence of the cluster of sporadic PSP in Wattrelos, France.

## Results

The disposal of contaminated waste in industrialized regions is associated with different medical conditions including neurodegenerative diseases^19^. The region of Wattrelos in northern France is highly industrialized. Thus, the French government and the French School of Advanced Studies in Public Health (*French école des hautes études en santé publique*) have directly assessed the environment in Wattrelos for contamination. Both studies concluded that the soil, water and the phosphate ores present in the industrial slag heaps in this region showed highly elevated levels of the heavy metals chromium, nickel and cadmium. The chronic exposure to Cr, Ni and Cd is toxic for humans and has been associated with different neurodegenerative diseases, including tauopathies such as Alzheimer’s disease^19–22^. However, little is known about the mechanisms by which these three heavy metals induce PSP pathogenesis and tau accumulation.

To understand the relationship between heavy metals and PSP, we analyzed the response of different cell models against exposure to the three heavy metals that accumulated in PSP patient environment: Cr (VI) (K_2_Cr_2_O_7_); Ni (II) (NiCl_2_) and Cd (CdCl_2_).

### Exposure to chromium and nickel induced neuronal death in iPSC-derived iNeurons carrying the R406W tau mutation

**Figure 1:**
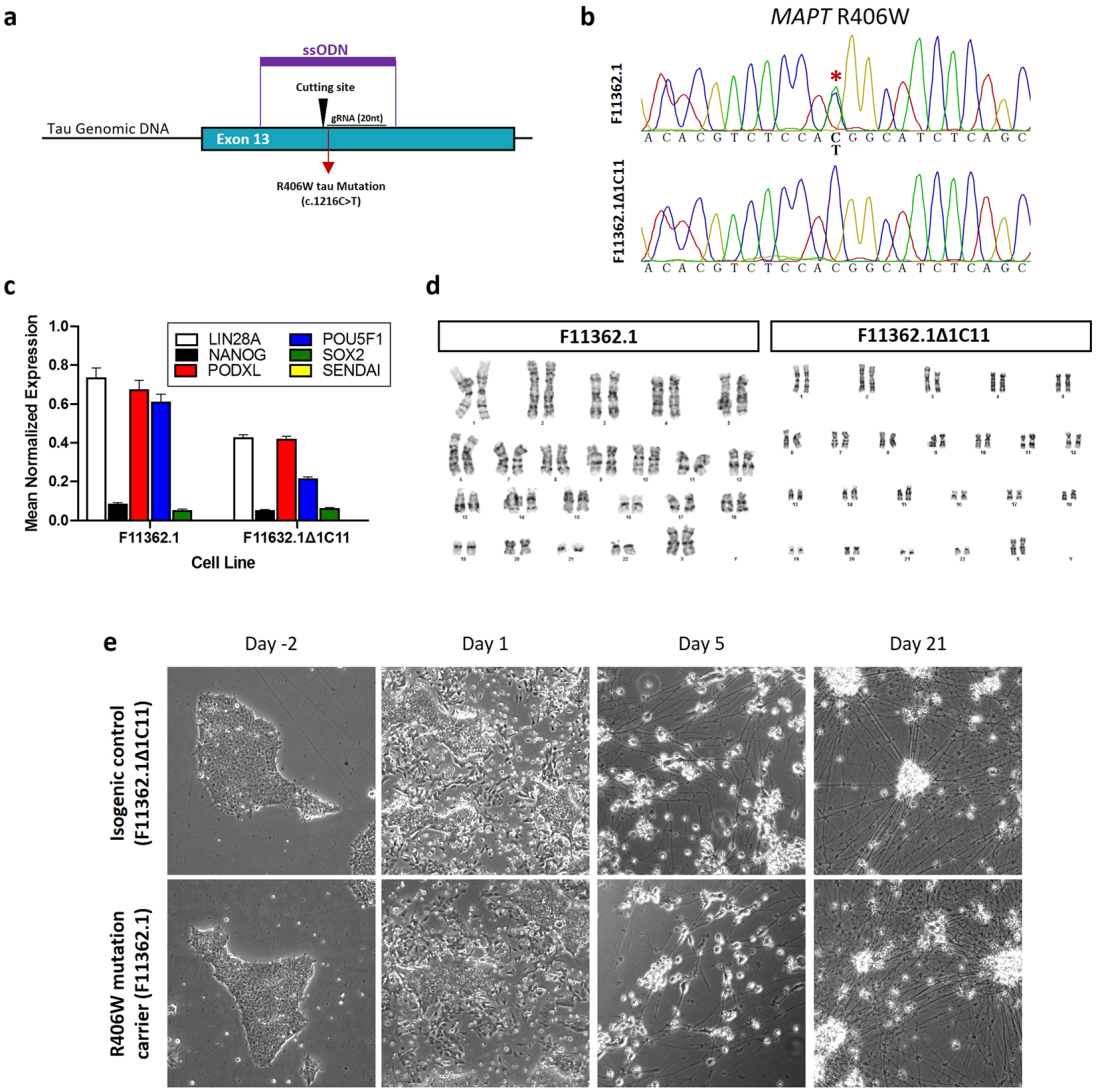
Genetic correction and characterization of the patient-derived iPSC lines. (**a**) Schematic representation of the CRISPR/Cas9 gene engineering protocol used to correct the mutant allele (c.1216 C > T) in a human iPSC line carrying the R406W tau mutation (F11362.1) to generate an isogenic control iPSC line (F11362.1Δ1C11). (**b)** Sanger sequencing confirming the presence of a c.1216 C > T substitution in a single allele of exon 13 in the *MAPT* gene corresponding to the R406W tau mutation (F11362.1) that was corrected in the isogenic control iPSC line (F11362.1Δ1C11). (**c)** qPCR showing the relative expression of pluripotency markers from the embryonic stem cell lines. *GAPDH* was used as gene of reference (**d)** G-band karyotyping showing that both R406W tau mutation carrier (F11362.1) and CRISPR/Cas9-corrected control lines (F11362.1Δ1C11) display no chromosomal abnormalities. (**e)** Representative microscopy images (10x) showing the neuronal differentiation of both R406W mutant and isogenic control iPSC lines. Cells presented neuronal phenotype 21 days after starting the differentiation process. No differences were found in cell viability and neuronal differentiation between isogenic control (F11362.1Δ1C11) and R406W tau mutant (F11362.1) iPSC lines.

Since both genetic and environmental risk factors contribute to the development of neurodegenerative diseases^23,24^, a highly relevant model for testing the neurotoxic effects of heavy metals is the use of iPSC-differentiated neurons carrying PSP-associated mutations. Therefore, we used an iPSC line (F11362.1) generated from an individual carrying a heterozygous mutation in the *MAPT* gene (R406W)^10^, which has been implicated in PSP and other tauopathies^8,9,25^. Since one of the limitations of using of patient-derived iPSCs is the lack of genetically paired controls, we used the CRISPR/Cas9 genome editing technology^10,26^ to generate an isogenic control iPSC line (Fig. 1a). We validated the successful genetic correction of the mutation by Sanger sequencing (Fig. 1b), and confirmed the pluripotency status of both R406W tau mutant and isogenic control iPSC lines by qPCR measuring the expression *LIN28A, NANOG, PODXL, POU5F1, SOX2* genes (Fig. 1c). Furthermore, using G-band karyotyping we confirmed that no chromosomal aberrations were introduced during the iPSC generation and the gene editing process (Fig. 1d).

**Figure 2:**
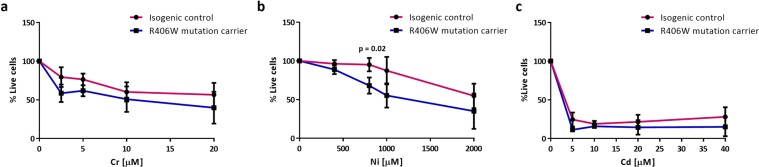
Cr and Ni treatments in iPSC-derived iNeurons 30,000 iPSC from a R406W mutation carrier individual (F11362.1) and isogenic control line (F11362.1Δ1C11) were seeded in triplicate in 96 well plates and then differentiated into iNeurons for 3 weeks. After differentiation, iNeurons were treated with increasing doses of chromium (0–20 µM) **(a)**, nickel (0–2000 µM) **(b)** and cadmium (0–40 µM) **(c)** for 72 hours and the (3‐(4,5‐dimethylthiazol‐2‐yl)‐2,5‐diphenyltetrazolium bromide) (MTT) assay was performed to assess the cell death induced by these three heavy metals. Results represent the percentage of live cells in treated iNeurons compared to untreated ones. Data shown are the mean ± SEM of 3 independent experiments for each heavy metal. Treatment with 800 µM Ni induced statistical significant cell death in R406W mutation carrier iNeurons compare with isogenic control (p-value < 0.05).

Using a standard protocol, we directly differentiated both isogenic control and R406W mutant iPSC lines into post-mitotic neurons (iNeurons)^27^ (Fig. 1e and Supplementary Fig. 1). The differentiated iNeurons were treated with increasing doses of Cr (0–20 µM), Ni (0–2000 µM) and Cd (0–40 µM) for 72 hours and the percentage of cell viability was measured by the MTT assay (Fig. 2). All three heavy metals caused a concentration-dependent cytotoxic effect indicated by the dose-dependent decrease in the percentage of live cells in both control and mutant lines (Fig. 2a–c and Supplementary Fig. 2). Interestingly, iNeurons carrying the R406W mutation tended to be more sensitive to cell death induced by chromium and nickel treatments than isogenic controls. Only the treatment with 800 µM of Ni induced a significant difference in the cell death between control and R406W tau mutant iNeurons (Fig. 2b and Supplementary Fig. 2). Surprisingly, cadmium treatment was equally and highly toxic for both control and *MAPT* mutant iNeurons (Fig. 2c)

To investigate whether the neurotoxic effect of these three heavy metals was related with the elevated concentration in the environment of Wattrelos, we treated control and R406W tau mutant iNeurons with aluminum (Al), a heavy metal not reported to be contaminating this region. Al did not induce cell death in either control or mutant iNeurons (Supplementary Fig. 3), suggesting that heavy metals accumulated in the environment of PSP patients may be associated with PSP-associated neuronal death.

Together these results indicated that R406W tau mutant iNeurons trended to be more sensitive to cell death induced by Cr and Ni, while Cd was equally toxic for both mutant and isogenic control iNeurons. Thus, we decided to further explore further the potential mechanisms underlying Cr and Ni neurotoxicity.

### Chromium and nickel induced cell death in cycling and terminally differentiated SH-SY5Y neuron-like cells

Since PSP is characterized in part by the loss of dopaminergic neurons in the midbrain^2^, we investigated whether heavy metal exposure affects this neuronal type. Therefore, we turned to SH-SY5Y cells, a neuroblastoma cell line that can be easily differentiated into dopaminergic neuron-like cells by the addition of retinoic acid (RA) and brain-derived neurotrophic factor (BDNF)^28^ (Supplementary Fig. 4). Because heavy metal toxicity in PSP preferentially affects neurons^29^, which are non-dividing post-mitotic cells^30^, we compared the sensitivity of SH-SY5Y cells to heavy metals in both non-differentiated and neuron-like differentiated states.

**Figure 3:**
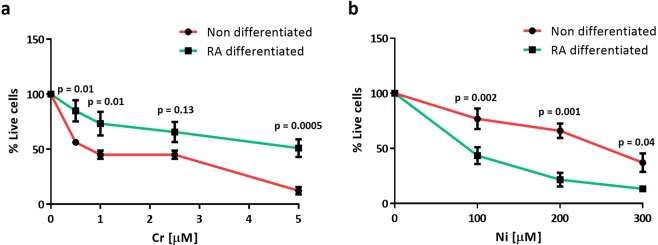
Non-differentiated SH-SY5Y cells are highly sensitive to Cr treatment but RA-differentiated ones are more vulnerable to Ni exposure. Non differentiated and RA-differentiated SH-SY5Y cells seeded in triplicate in 96 well plates and were treated with increasing concentrations of Cr (0–5 µM) **(a)** and Ni (0–300 µM) **(b)** for 24 hours. Plots represent the percentage of live cells after heavy metal treatments relative to untreated ones determined using the MTT assay. Data show the mean ± SEM of 5 independent experiments. Statistical significance was determined by two‐way analysis of variance (anova) followed by Bonferroni’s test for multiple comparisons using GraphPad Prism 6. p-values comparing non-differentiated vs RA-differentiated cells after Cr and Ni treatments are included in the graphs. Statistical significance was considered when p-values were minor or equal to 0.05.

Non-differentiated and RA-differentiated SH-SY5Y cells were treated with increasing doses of Cr (0–5 µM) and Ni (0–300 µM) for 24 hours and MTT assays were performed to determine cell death. Cr and Ni treatments induced significant cell death in a dose-dependent manner in both differentiated and non-differentiated cells (Fig. 3 and Supplementary Fig. 5). Interestingly, exposure to Cr and Ni differentially affected the cell viability of RA-differentiated and non-differentiated cells. Non-differentiated cells were more sensitive to Cr treatment whereas RA-differentiated cells were more sensitive to Ni exposure (Fig. 3). The divergent effects of Cr and Ni exposure on dividing and non-diving cells suggests that Ni exposure may preferentially affect post-mitotic cells and/or dopaminergic neuron-like cells.

### Chromium and nickel treatments increased tau protein levels and phosphorylation in iPSC-iNeurons and SH-SY5Y cells

**Figure 4:**
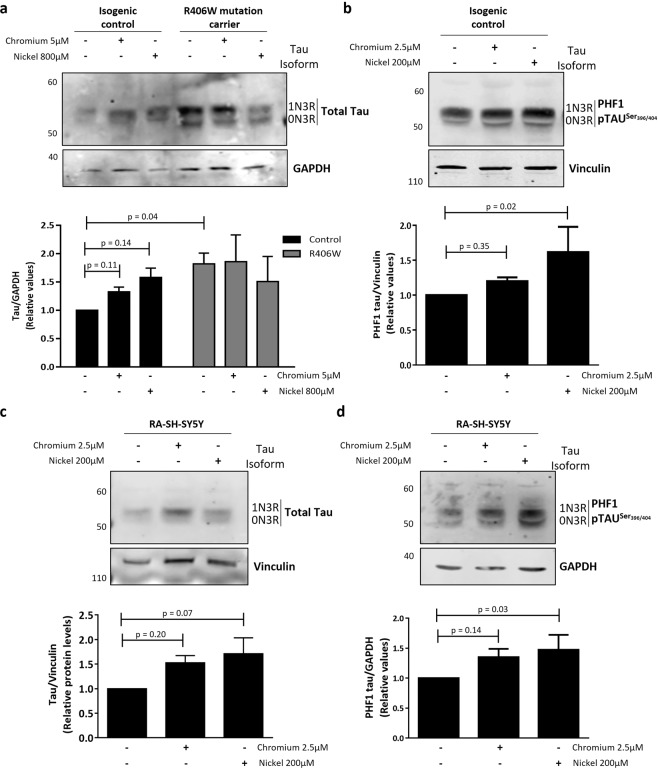
Cr and Ni exposure increases tau levels and phosphorylation in iNeurons and RA-differentiated SH-SY5Y cells. (**a**,**b**) iPSCs carrying the R406W tau mutation and isogenic controls were differentiated into iNeurons and treated with Cr (5 µM) and Ni (800 µM) for 72 hours. The levels of total **(a)** and phospho-tau^Ser396/404^ (PHF-1) **(b)** tau were measured by western blot. GAPDH and vinculin were used as loading controls. Images show representative immunoblots comparing tau and phospho-tau levels in control and mutant iNeurons before and after Cr and Ni treatment. Plots represents the average ± SEM of 3 independent experiments. (**c**,**d**) RA-differentiated SH-SY5Y cells were treated with Cr (2.5 µM) and Nickel (200 µM) for 24 hours before protein extraction. Images show representative immunoblots comparing the levels total tau **(c)** and phospho-tau^Ser396/404^ (PHF-1) tau **(d)** before and after heavy metals treatment. GAPDH and vinculin were used as loading control. Plots represent the average ± SEM of 4 independent experiments. Statistical significance was determined by two‐ANOVA followed by Bonferroni’s test for multiple comparisons or one-way ANOVA using GraphPad Prism 6. p-values comparing untreated and heavy metal treated cells are included in the graphs. Statistical significance was considered when p-value ≤ 0.05.

The neuropathology of PSP subjects demonstrate inclusions of hyperphosphorylated tau in affected neurons. To determine if exposure to heavy metals could increase tau pathology *in vitro*, we assessed the levels of total and phosphorylated tau protein before and after heavy metal exposure in iNeurons and neuron-like SH-SY5Ycells. iNeurons carrying the R406W tau mutation in *MAPT* and paired isogenic control lines were exposed to Cr (5 µM) and Ni (800 µM) for 72 hours (Fig. 4a,b). These doses were selected based on greatest differential in cell death induced by these treatments between R406W mutant iNeurons and its isogenic control. Cells were collected post-treatment, and the levels of total and phosphorylated tau were determined by western blot. In control iNeurons, exposure to both Cr and Ni resulted in increased levels of total tau and phosphorylated tau. In contrast, while R406W tau mutant iNeurons displayed significantly higher baseline levels of tau protein compared with the isogenic controls, Cr and Ni treatments did not further increase total tau protein levels (Fig. 4a,b and Supplementary Figs. 6 and 8). Taken together these results showed that Cr and Ni caused an increase in tau levels similar to the levels observed in iNeurons carriers of the tau mutation, thus “mimicking” the known effect of the R406W tau variant, known to be the cause of tauopathy. Next, we determined if the heavy metal-induced increase in tau levels and phosphorylation observed in iNeurons also occurred in RA-differentiated SH-SY5Y cells. Cells were treated for 24 hours with 2.5 µM Cr and 200 µM Ni and then levels of tau were determined by western blot. These doses were chosen as they showed statistically significant cell death in differentiated SH-SY5Y cells (Supplementary Fig. 5). Similar to the results found in control iNeurons, both heavy metals increased total and phosphorylated tau levels in RA-differentiated neuron-like SH-SY5Y cells (Fig. 4c,d and Supplementary Figs. 7 and 8). The elevation in tau levels associated with Cr and Ni exposure could be due to an increase of tau expression or a defect in tau degradation. To assess this, we measured *MAPT*/tau mRNA levels in RA-differentiated SH-SY5Y cells before and after Cr and Ni exposure. Ni treatment didn’t affect *MAPT*/tau mRNA levels. Surprisingly, although tau protein levels are elevated after Cr exposure, treatment with this heavy metal decreased *MAPT*/tau expression suggesting a compensatory mechanism (Supplementary Fig. 9).

Together our data indicate that the exposure to Cr and Ni may induce a failure in tau protein degradation leading to the accumulation of hyperphosphorylated forms of tau in neuronal-like cell models.

### Heavy metal treatment induced apoptosis in neuroblastoma cell lines

**Figure 5:**
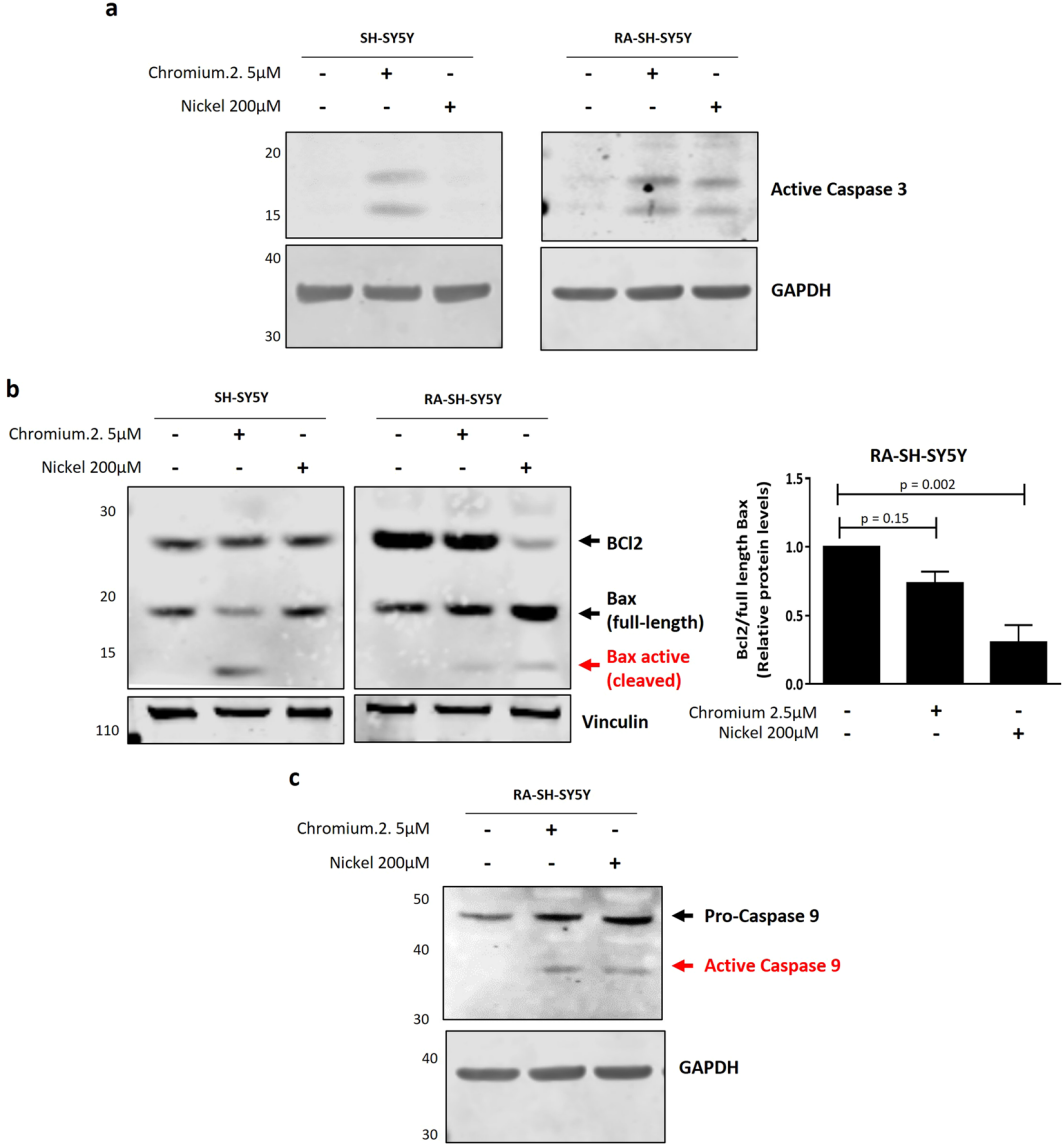
Cr and Ni exposure induces apoptotic cell death in SH-SY5Y cells. Non-differentiated and RA-differentiated SH-SY5Y cells were treated with Cr (2.5 µM) and Ni (200 µM) for 24 hours. Whole cell lysates were collected in order to analyze by western blot the levels of cleaved caspase-3 protein **(a)**, the ratio of anti-apoptotic protein Bcl2 to pro-apoptotic protein Bax **(b)** and the cleavage and activation of caspase-9 **(c)**. Vinculin and GAPDH were used as loading controls. (**a)** Representative immunoblot showing the presence of the 17/19KD fragment of caspase-3 after Cr and Ni exposure. (**b)** Representative immunoblots showing the levels of Bcl2 and Bax proteins after heavy metal exposure. The activation of the intrinsic/mitochondrial apoptosis pathway was assessed by the decrease of the ratio of Bcl2 to full length Bax and the presence of a cleavage form of Bax protein. The plot represent the average ± SEM of 3 independent experiments. Statistical significance was determined by one-way analysis of variance (anova) followed by Bonferroni’s test for multiple comparisons using GraphPad Prism 6. p-values comparing untreated and heavy metal treated cells are included. Statistical significance was considered when p-value ≤ 0.05. (**c)** Immunoblot showing the presence of the fragment (43KD) of active caspase-9 after Cr and Ni exposure to confirm the activation of the intrinsic/mitochondrial pathway. All experiments were performed in triplicates.

Our previous results showed that Cr and Ni treatments induced neurotoxicity in both iNeurons and SH-SY5Y neuron-like cells (RA-differentiated and non-differentiated). Cell death can occur via multiple mechanisms^31^. Apoptosis is a highly regulated type of programed cell death characterized by morphological and biochemical events leading to the cleavage and activation of the executioner caspase-3^31^. Alterations in the apoptotic pathway have been associated with neurodegeneration^32–35^, and a variety of environmental toxins including heavy metals have been reported to induce apoptosis^36–38^. Therefore, we asked if Cr and Ni exposure induced cell death by an apoptotic mechanism. To study apoptosis, first we assessed the activation of caspase-3 measuring the levels of cleaved caspase-3 protein (17/19 kD fragment). Our results showed that Cr (2.5 µM) treatment induced caspase-3 activation in non-differentiated and differentiated cells (Fig. 5a). However, Ni (200 µM) exposure induced the activation of caspase-3 only in RA-differentiated SH-SY5Y cells, suggesting that RA differentiation sensitizes SH-SY5Y cells apoptotic cell death induced by Ni (Fig. 5a and Supplementary Fig. 11). Apoptosis can be initiated by two independent pathways: the intrinsic or mitochondria-dependent (intrinsic/mitochondrial) and the extrinsic or mitochondria-independent. The intrinsic/mitochondrial pathway starts in response to an intracellular stress that modulates the activity of two proteins of the Bcl family: Bcl2 (anti-apoptotic) and Bax (pro-apoptotic). The activation of Bax leads to the release of cytochrome c from the mitochondria and the cleavage and activation of the pro-apoptotic protein caspase-9 (Supplementary Fig. 10). To assess if Cr and Ni exposure activated apoptosis by the intrinsic/mitochondrial pathway, we measured the activation of caspase-9 as well as the ratio of Bcl2 to Bax^39,40^. A decrease in the Bcl2/Bax ratio and/or the cleavage of the Bax protein indicates that the intrinsic/mitochondrial apoptotic pathway is activated. Our results showed that Ni and Cr exposure decreased Bcl2/Bax ratio and cleaved caspase-9 in RA-differentiated SH-SY5Y, indicating that these heavy metals induced apoptosis by the intrinsic/mitochondrial mechanism in SH-SY5Y cells (Fig. 5b,c and Supplementary Fig. 12). Interestingly, Cr treatment induced intrinsic/mitochondrial apoptosis more robustly in non-differentiated SH-SY5Y cells compared with RA-differentiated, as was shown by the cleavage of the pro-apoptotic protein Bax (Fig. 5b and Supplementary Fig. 10). Data presented here supports our previous results showing that Ni neurotoxicity was more evident in RA-differentiated SH-SY5Y cells, but Cr was more toxic in non-differentiated cells (Fig. 3).

Together, these results demonstrate that Cr and Ni exposure induces intrinsic/mitochondrial apoptosis and that the differentiation of SH-SY5Y cells affects the neurotoxicity induced by Cr and Ni.

## Discussion

PSP is a neurodegenerative disease with a predominantly tau neuropathology^41^. Although PSP etiology is not completely understood, genetic^6^ and epigenetic^42^ variants have been reported to contribute to the risk for PSP. Moreover, circumstantial evidence suggests that continued exposure to environmental toxins, including heavy metals, could increase the risk of PSP^11–14,18,19^.

Recently, a non-familial PSP cluster has been identified in a region in the north of France home to metal-related industries that improperly deposit ore waste in residential areas^15^. Here, we have investigated the neurotoxicity mechanism associated with chromium, nickel and cadmium, three heavy metals contaminating the region where the non-familiar PSP cluster was identified. These three heavy metals are widely used in industry, extremely toxic for humans and have been associated with the development of neurodegenerative diseases^19,22,43,44^. Therefore, we hypothesized that the chronic exposure to Cr, Ni and Cd could be related to the presence of the sporadic PSP cluster in Wattrelos, France.

To better understand the mechanisms underlying neurotoxicity associated with Cr, Ni and Cd exposure, we have studied the effects induced by those metals in iPSC-iNeurons and SH-SY5Y neuron-like cells. Although toxicity studies of these elements have previously been addressed in cultured cells^45–49^, this is the first time that a comparative study of heavy metal neurotoxicity was performed using iPSC-derived iNeurons carrying the PSP-associated R406W tau mutation.

Despite some disadvantages, such as the high cost of iPSC generation and a time and labor intensive protocol for neuronal differentiation, human iPSC-derived neurons play an important role in disease modeling and are used to study neurotoxicity of environmental contaminants^50^ or chemotherapeutic drugs^51–53^. Limiting the utility of iPSCs generated from disease-mutation carriers is the lack of isogenic unaffected controls. Most current studies use age-matched unaffected cells within the same family pedigree as controls^54,55^, but these are suboptimal due to the intra-family differences in the genetic background. To avoid this, an innovative strategy is to use the CRISPR/Cas9 genome editing technology to generate isogenic controls by correcting disease-causing mutations^56^. Here, we corrected the R406W tau mutant allele in the patient-derived iPSC line to obtain an isogenic control iPSC line that represent a unique tool for studying the impact of genotype on neurotoxicity induced by exogenous toxins, in this case heavy metals.

iNeurons carrying the R406W variant in tau were tend to be more vulnerable than isogenic control iNeurons to the toxicity induced by both Cr and Ni. The double-hit hypothesis of neurodegeneration proposes that neurodegenerative diseases are caused by the accumulation of genetic and environmental risk factors^23,24,57^. Thus, we hypothesize that Cr and Ni may accelerate neuronal death in individuals carrying genetic variants that increase the risk of PSP. Although Cd treatment was highly toxic for both control and R406W tau mutant iNeurons, Cd concentration was lower than the concentration of Ni and Cr in Wattrelos environment. On the other hand, the treatment with aluminum, a heavy metal not enriched in the environment of Wattrelos, did not induce cytotoxicity in iNeurons. Together, these results suggest that Cr and Ni exposure, but not other heavy metals such as Al, could be potentially associated with the sporadic PSP cluster found in Wattrelos and support previous studies suggesting that Al and Cd exposure is associated with some neurodegenerative diseases such as AD, but not with PSP^19,58,59^.

We also studied the cell death induced by chromium and nickel in SH-SY5Y neuroblastoma cells, a well-known dopaminergic neuronal-like cell model previously used to study neurotoxicity^60,61^. This cell line is characterized by the easy differentiation of the cycling cells into neuronal-like cells by the treatment with RA and BDNF^28^. Both heavy metals induced cell death in SH-SY5Y cells in a dose-dependent manner, but we found that their effects differed in that RA-differentiated SH-SY5Y cells were more sensitive to Ni but more resistant to Cr treatment than non-differentiated SH-SY5Y cells. It has been described that RA-mediated differentiation affects the response of SH-SY5Y neuroblastoma cells to neurotoxins^61^. Moreover, RA treatment of SH-SY5Y cells affects mitochondria and energy metabolism^62–64^, making differentiated neuron-like cells more resistant to the oxidative stress induced by heavy metals^37,38,65^. Although more studies are needed to understand why RA treatment makes SH-SY5Y cells more sensitive to Ni and more resistant to Cr, these two heavy metals may induce neuronal death by different mechanisms. Cr and Ni have opposite effects in the regulation of the PI3K/AKT survival signaling pathway, Cr treatment inhibits PI3K/AKT activity^66^ while Ni exposure activates the pathway^67^. RA treatment induces the activation of survival pathways including PI3K/AKT in SH-SY5Y cells^61^, thus we speculate that the addition of RA could be protecting the neuron-like differentiated SH-SY5Y cells from toxicity induced by Cr but sensitizing them to the cell death induced by Ni.

Interestingly, our results showed that SH-SY5Y cells and iPSC-derived neurons have distinct responses to heavy metal treatments. Overall, iNeurons were more resistant to heavy metals than SH-SY5Y cells. iNeurons showed cell death after 72 hours of heavy metal treatment while only 24 hours of Cr and Ni exposure was enough to induce toxicity in SH-SY5Y cells. The contrasting responses to heavy metals-induced toxicity of SH-SY5Y cells and iNeurons may be explained by the differences in the neuronal population between these two cell lines. Differentiated SH-SY5Y cells present a homogeneous population of dopaminergic neuron-like cells^28^, but the iNeurons generated here are a heterogeneous population of cortical neurons containing mostly GABAergic and glutamatergic neurons^27^. PSP is characterized in part by the loss of dopaminergic neurons of the substantia nigra^2^. Therefore, our observation that the SH-SY5Y dopaminergic neuron-like cells were highly sensitive to the toxicity induced by Cr and Ni supports the idea of that exposure to heavy metals increase tau accumulation and potentially the risk of PSP.

We found that Cr and Ni exposure induces cell death by an overactivation of the mitochondrial/intrinsic apoptosis pathway. This is in agreement with previous reports showing that both heavy metals promote mitochondrial/intrinsic apoptosis by the alteration in Bcl2/Bax ratio, mitochondrial instability, release of cytochrome c and caspase-9 activation^37,39,43,68–71^. Moreover, our finding that treatment with neurotoxic doses of Cr and Ni increased the levels and phosphorylation status of tau protein in both iNeurons and RA-differentiated SH-SY5Y cell lines suggests that tau pathology could be an intermediate step in neuronal death induced by these heavy metals. Although further investigation should be performed in order to understand the mechanisms underlying heavy metal-related tau accumulation and phosphorylation, our results supports previous work demonstrating increased tau pathology in populations high exposed to Cr and Ni^72^. The results presented here suggest that heavy metals exposure could potentially increase the risk of tauopathies such as PSP.

In summary, our results showed that the exposure to three heavy metal (Cr, Ni and Cd) contaminating a geographic region associated with a cluster of sporadic PSP patients induced dose-dependent neurotoxicity *in vitro*. Interestingly, iNeurons carrying the PSP-related tau variant R406W trended to be more sensitive to the cell death induced by Cr and Ni. Although more experiments need to be performed to understand how heavy metals induce neurodegeneration, these results suggest that the exposure to some environmental toxins may accelerate neural death in PSP-related mutations carriers. Strikingly, RA-differentiated SH-SY5Y neuron-like cells showed distinct tolerance to Cr and Ni-induced apoptotic cell death as compared to non-differentiated cells, suggesting that these two heavy metals induce apoptosis through different mechanisms. Additionally, we showed that neurotoxic doses of Cr and Ni increased the levels and phosphorylation status of tau protein *in vitro*, linking heavy metal exposure with the development of tauopathies such as PSP. In conclusion, the current work suggests that the risk of tau-related neurodegenerative diseases including PSP may be elevated in areas contaminated by certain heavy metals such as chromium and nickel, and that prevention or mitigation of such contamination may reduce the population risk of these disorders.

## Experimental Procedures

### Heavy metals

The following heavy metals were used in this study: potassium dichromate (K2Cr2¬O7) (Sigma, #7778-50-9, 10 mM stock), nickel (II) chloride (Sigma, #7718-54-9, 1 M stock) and cadmium chloride (CdCl2) (Sigma, #10108-64-2, 100 mM stock). Heavy metal stock solutions were all dissolved in ultrapure water using 18.2 mega-ohm-cm water from a Milli-Q purification system (formerly Millipore, Billerica, MA now MilliporeSigma, Burlington, MA) and further diluted in designated media as necessary.

### Cell lines

SH-SY5Y neuroblastoma cells (ATCC, #CRL-2266), were cultured in EMEM:F12 medium supplemented with 10% (v/v) heat-inactivated fetal bovine serum (FBS) and 1% penicillin/streptomycin following the instructions provided by ATCC.

iPSC lines were maintained in mTSER media (StemCell technologies). Upon 80% confluence, cells were detached from the plate using accutase (StemCell technologies) and seeded onto matrigel (Corning Bioscience) coated plates using mTSER media supplemented with 10 µM Rock inhibitor (Y-27632, StemCell technologies). Media was replaced every day.

### Genome engineering and characterization of the iPSC lines

The iPSC line (F11362.1) was generated from epithelial fibroblasts from a heterozygous R406W tau mutation carrier. The skin biopsy was performed after obtaining the donor’s written informed consent^10^. The informed consent and the protocol of our study was approved by the Washington University School of Medicine Institutional Review Board and Ethics Committee (IRB 201104178 and 201306108). The consent allowed for use of tissue by all parties, commercial and academic, for the purposes of research but not for use in human therapy. All the experimental protocols and methods were performed in accordance with the guidelines and regulations of the Washington University School of Medicine Institutional Review Board and Ethics Committee.

#### CRISPR/Cas9 correction of the R406W mutation to generate an isogenic control iPSC line

Gene-corrected isogenic control line was generated using the CRISPRs/Cas9 system as previously reported^10,26^. Briefly, CRISPRs guides (sgRNA) targeting the mutant allele in exon 13 of the MAPT gene were designed to have at least 3 bp of mismatch to any other gene in the human genome and validated for activity using the T7E1 assay. Human iPSC were co-nucleofected with 1 μg gRNA, 3 μg Cas9 and 300 μM single stranded oligodeoxynucleotides (ssODN) using the P3 Primary Cell 4D reaction mix (Lonza)^10^. To select the iPSC lines presenting the corrected R406W mutation, genomic DNA was extracted following the QuickExtract protocol (Epicentre) and PCR was performed using Q5 Hot Start High-Fidelity 2X Master Mix. The primers used were GAGCAAGACCCTGTCTCAAA and ATTAACCGAACTGCGAGGAG. The PCR program was 98 °C for 30 s and 30 cycles of 98 °C for 10 s, 65 °C for 30 s, and 72 °C for 25 s and a cycle at 72 °C for 2 minutes. One CRISPR-corrected line (F11362.1Δ1C11) was identified, expanded, frozen and used as isogenic control.

#### Quantitative PCR analysis of pluripotency markers

Both R406W tau mutant and CRISPR-engineered isogenic control iPSC lines were verified to express pluripotency markers by qPCR as previously described^10^. Briefly, RNA was extracted from cell pellets with the RNeasy kit (Qiagen) following the manufacturer’s protocol. Extracted RNA (10 µg) was converted to cDNA by PCR using the High-Capacity cDNA Reverse Transcriptase kit (Life Technologies). The expression of pluripotence genes (SOX2, POU5F1, LIN28A, NANOG, SENDAI, PODXL) was measured by qPCR as previously described^73^. Primers specific to GAPDH were used as a loading control.

#### Karyotyping

Chromosomal abnormalities in both R406W mutant and the isogenic control iPSC lines were assessed by G-band karyotyping using standard cytogenetic procedures.

### iPSC differentiation

Human iPSC were differentiated into cortical neurons following a protocol previously described^27^. Briefly, 15,000 to 30,000 cells/cm^2^ were seeded in matrigel coated wells. 24 hours after seeding, iPSC were infected with virus expressing neurogenin-2 (Ngn2) and reverse tetracycline-controlled activator (rtTA) (Supplementary Fig. 1). The following day, the expression of Ngn2 was induced by the addition of 2 µg/mL doxycycline to N2 medium (1:1 EMEM/F12 medium supplemented with 1X N2, 1X NEAA, and supplemented with 10 ng/mL BDNF, 10 ng/mL NT3, 0.2 µg/mL mouse laminin) for 24 hours. Then, Ngn2-expressing cells were selected by the addition of 1 µg/mL puromycin. After selection cells were cultured in N3a medium (equal parts Neurobasal-A medium and 1:1 EMEM/F12 media supplemented with 0.5X B27, 0.5X N2, 0.75X GlutaMAX, 1X P/S, 0.5X NEAA, 0.35% (v/v) beta-mercaptoethanol). The N3a media was conditioned 24 h in mouse astrocytes culture prior to being added to iPSC-neuron cultures. Half the volume of N3a media was replaced every three days. On day 21, neurons were considered mature, and heavy metal compounds were added for 72 hours (Supplementary Fig. 1).

### SHSY5Y differentiation into neuron-like cells

SH-SY5Y cells were seeded in the corresponding plates at a density of 10,000 cells/cm^2^. The next day, 10 µM of retinoic acid (RA) was added to start the differentiation in EMEN/F12 media supplemented with 10% of FBS and 1% Penicillin-Streptomycin. After 6 days of RA treatment, cells were treated for 4 days with 50 ng/mL of BDNF in EMEN/F12 media supplemented only with 1% Penicillin-Streptomycin^28^ (Supplementary Fig. 2).

### Assessment of cell viability via MTT Assay

For the MTT assay, cells were seeded in triplicate in 96 well plates. After heavy metal treatments 10 µL of 5 mg/mL 3-(4,5-dimethylthiazol-2-yl)-2,5 diphenyltetrazolium bromide reactive (MTT) was added to each well containing 100uL of media and incubated for 4 h at 37 °C. Active mitochondria of living cells can cleave MTT to produce formazan, a purple crystal whose level is directly proportional to the number of living cells. After incubation, formazan crystals were solubilized in 100uL DMSO and absorbance was measure at 595 nm.

### Quantitative PCR analysis of *MAPT*/tau levels

RA-differentiated SH-SY5Y cells were treated with Cr (2.5 µM) and Ni (200 µM) for 24 hours. Then, cells were harvested and mRNA was extracted from cell pellets with the RNeasy kit (Qiagen) following the manufacturer’s protocol. Extracted mRNA was converted to cDNA by PCR using the High-Capacity cDNA Reverse Transcriptase kit (Life Technologies). The expression of *MAPT*/tau gene was measured by qPCR as previously described^73^. *GAPDH* were used as housekeeping gene.

### Western blotting

Cells treated with Cr and Ni were harvested and washed with phosphate buffered saline (PBS). Cells were lysed with Pierce radioimmunoprecipitation (RIPA) buffer (25 mM Tris•HCl, pH 7.6, 150 mM NaCl, 1% NP-40, 1% sodium deoxycholate and 0.1% SDS) containing mini-complete protease and phosphatase inhibitor cocktails (Roche). Lysates were centrifuged at 15,000 rpm for 15 min at 4 °C to obtain the supernatant containing soluble proteins. Protein concentration was determined by bicinchoninic acid (BCA) assay (Thermo). Equivalent amounts of protein were separated on 4–12% SDS-PAGE (Thermo), then transferred onto nitrocellulose membranes (Bio-Rad). Membranes were blocked at room temperature with Odyssey Blocking Buffer (LI-COR 927-40100) and incubated at 4 °C overnight with primary antibodies and 1 hour at room temperature with appropriate fluorescent secondary antibodies (1:5,000) (LI-COR). Immunoreactive bands were visualized using a LI-COR Odyssey CLx image scanner and quantified using ImageJ software. The following primary antibodies were used: monoclonal mouse anti-human tau HT7 (Thermo Scientific, #MN1000, 1:250); monoclonal mouse anti-tau phospho-Ser396/Ser404 (PHF-1) (Peter Davies lab, Litwin Zucker Center for Alzheimer’s Research, Long Island, USA); monoclonal mouse anti-GAPDH (Abcam, #ab8245, 1:1,000); monoclonal mouse anti-vinculin (R&D MAB6896, 1:500). monoclonal mouse anti-Bcl2 (Cell signaling technologies, #15071, 1:500), rabbit monoclonal anti-Bax (Cell signaling technologies, #5023, 1:500), mouse monoclonal anti-caspase-9 antibody (Cell signaling technologies, #9508, 1:500) and monoclonal rabbit anti-caspase-3 (D3R6Y) (Cell signaling technologies, #14220, 1:500).

### Statistical analysis

One-way and two-way ANOVA statistical analyses were performed using Prism GraphPad Prism 6. Bonferroni’s analysis was performed to analyze the statistical significance between the groups. Plots show means ± standard error (SEM) of all the experiments performed. Values are considered statistical significant if p < 0.05.

## Supplementary information

Supplementary figures.

## Data Availability

The authors confirm that the data supporting the findings of this study are available within the article [and/or] its Supplementary Materials.
