# Supplementary material for: Heavy metals contaminating the environment of a progressive supranuclear palsy cluster induce tau accumulation and cell death in cultured neurons
Source: Sci Rep. 2020 Jan 17;10:569. doi: 10.1038/s41598-019-56930-w (PMC6969162; doi:10.1038/s41598-019-56930-w)
Supplement: Supplementary file 1 — Supplementary figures. [file 41598_2019_56930_MOESM1_ESM.pdf]

Supplementary Data:

Heavy metals contaminating the environment of a progressive supranuclear palsy cluster induce tau accumulation and cell death in cultured neurons

Carolina Alquezar[1], Jessica B. Felix[2], Elizabeth McCandlish[3], Brian T. Buckley[3], Dominique Caparros-Lefebvre[4], Celeste M. Karch, Lawrence I. Golbe[3], Aimee W. Kao[1]*.

[1]Memory and Aging Center, Department of Neurology, University of California, San Francisco, California, [2]Graduate Program, Department of Molecular and Cellular Biology Baylor College of Medicine, Houston, Texas. [3]Rutgers Robert Wood Johnson Medical School, New Brunswick, [4]Centre Hospitalier de Wattrelos, France. [5]Department of Psychiatry, Washington University in St Louis, St Louis MO. *Corresponding author

Supplementary Figure 1

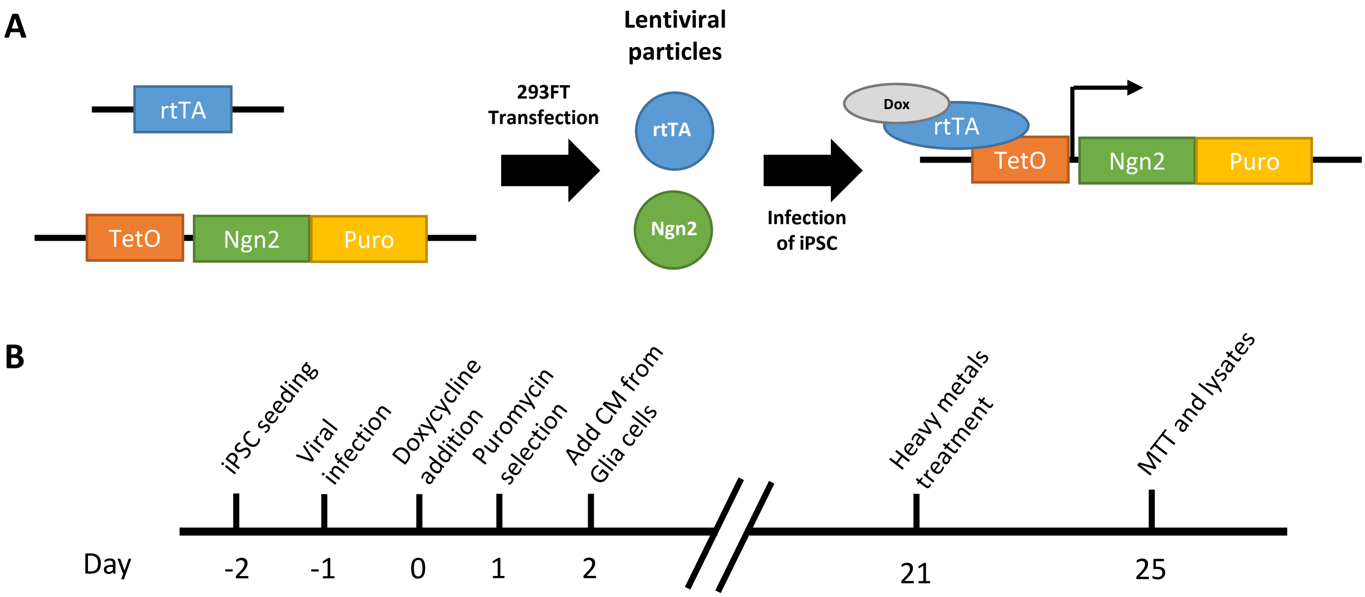

Supplementary Figure 1. Schematic representation of iPSC-iNeurons differentiation.

A) Lentiviral particles containing plasmids for the expression of rtTA and TetO-Nng2-Puro were made in HEK293FT cells. Then R406W tau mutant cells and isogenic controls were infected with both lentivirus to induce the expression of Ngn2 gene and start the neuronal differentiation process. **B)** Summary of the protocol used for the generation of iPSC-differentiated iNeurons and heavy metal treatments.

Supplementary Figure 2

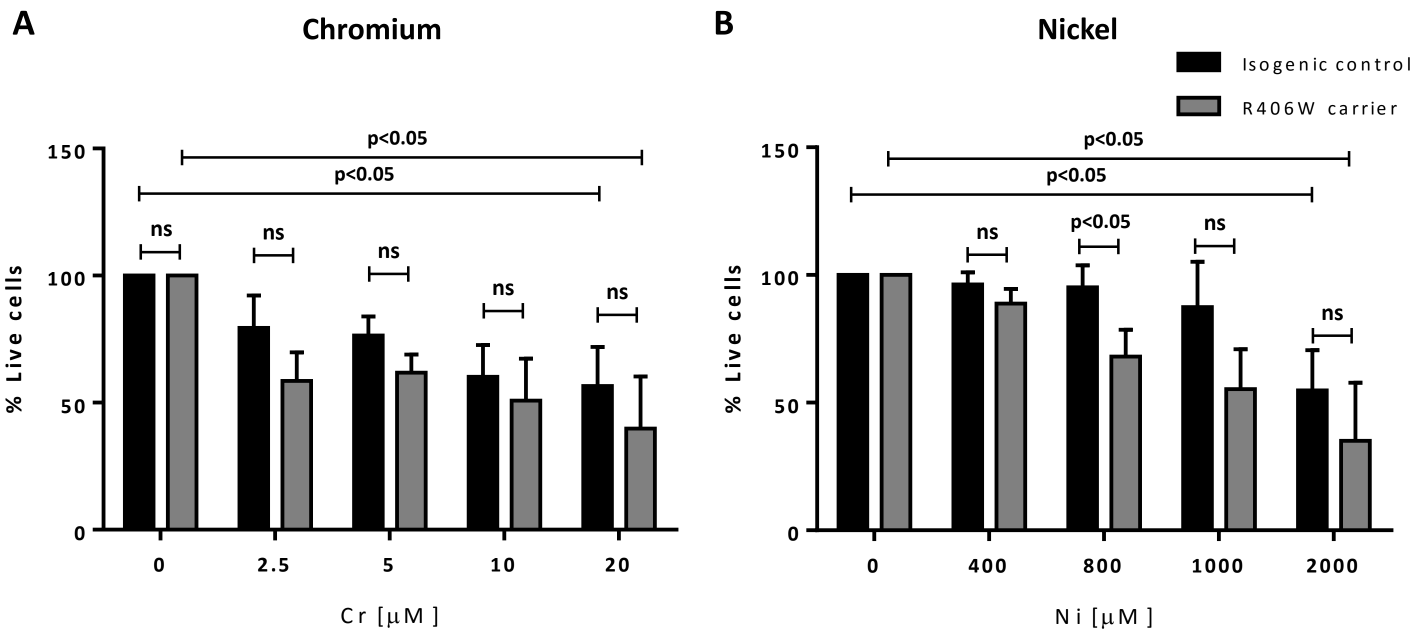

Supplementary Figure 2. Statistical analysis of the survival assays after Cr and Ni treatment in iNeurons.

A) Average ± SEM of the percentage of live cells after Cr treatment from 3 independent experiments. The Statistical significance was determined by the two-way analysis of variance (ANOVA) followed by Bonferroni's test for multiple comparisons using GraphPad Prism 6. Only 20µM Cr treatment induced significant cell death ($p<0.05$) in both isogenic control and R406W tau mutant iNeurons. There is no statistical significant differences in the cell death induced by Cr between control and R406W mutant iNeurons. **B)** Plot showing the mean ± SEM of the percentage of live cells after treatment with increased doses of Ni from 3 independent experiments. The statistical significance was determined by the two-way analysis of variance (ANOVA) followed by Bonferroni's test for multiple comparisons using GraphPad Prism 6. 2000µM Ni treatment induced significant cell death ($p<0.05$) in both isogenic control and R406W tau mutant iNeurons. Cell death induced by 800µM treatment is significantly different between control and R406W mutant iNeurons ($p<0.05$).

Supplementary Figure 3

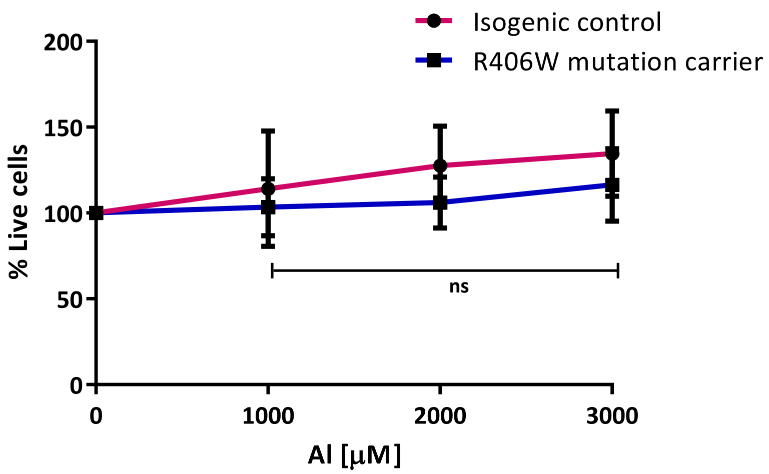

Supplementary Figure 3. Aluminum treatment did not induce cell death in both control and R406W iNeurons

iPSC from a R406W mutation carrier individual (F11362.1) and isogenic control line (F11362.1Δ1C11) were seeded in triplicate in 96 well plates and then differentiated into iNeurons for 3 weeks. After differentiation, iNeurons were treated with increasing doses of aluminum (0-3000µM) for 72 hours and the (3-(4,5-dimethylthiazol-2-yl)-2,5-diphenyltetrazolium bromide) (MTT) assay was performed to assess the cell death. Results represent the % of live cells of treated iNeurons relative to untreated ones. Data shown are the mean ± SEM of 3 independent experiments. The statistical significance was determined by two-way analysis of variance (ANOVA) followed by Bonferroni's test for multiple comparisons using GraphPad Prism 6. No cell death was observed after Aluminum treatments in both isogenic control and R406W tau mutant iNeurons.

Supplementary Figure 4

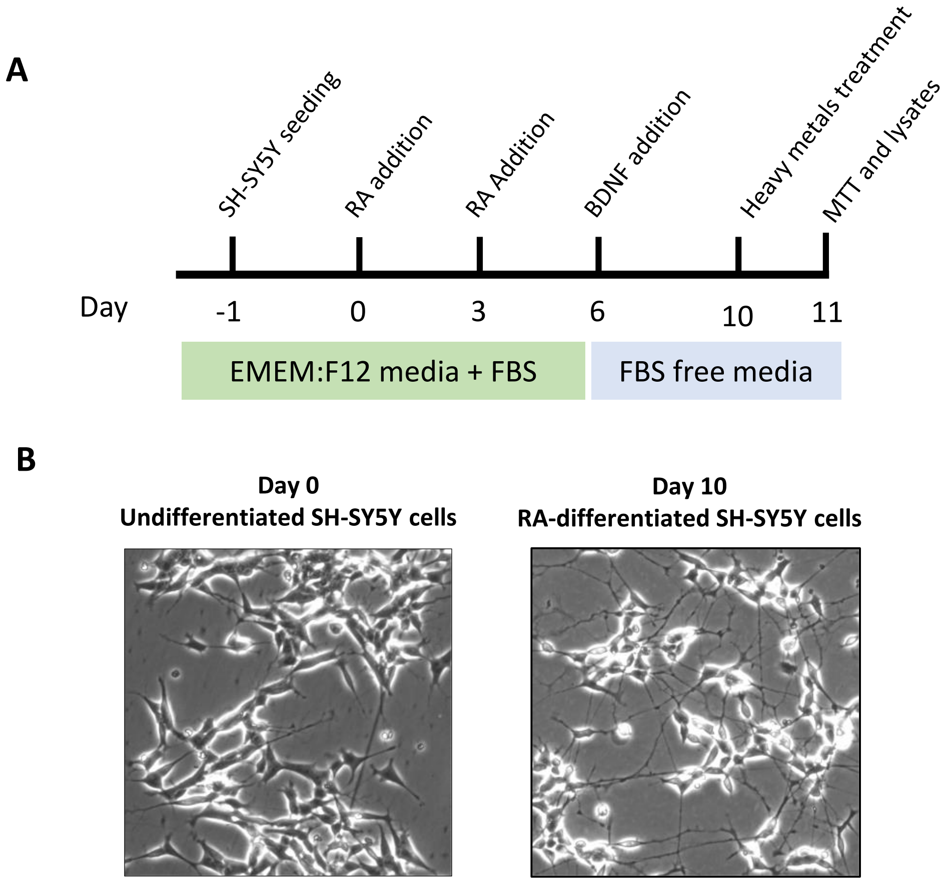

Supplementary Figure 4. Schematic representation of RA-differentiation of SH-SY5Y cells.

A) Diagram showing the protocol used for the differentiation of the SH-SY5Y cells and the heavy metal treatments. **B)** Representative pictures showing the SH-SY5Y cells before retinoic acid addition and at the completion of the differentiation process.

Supplementary Figure 5

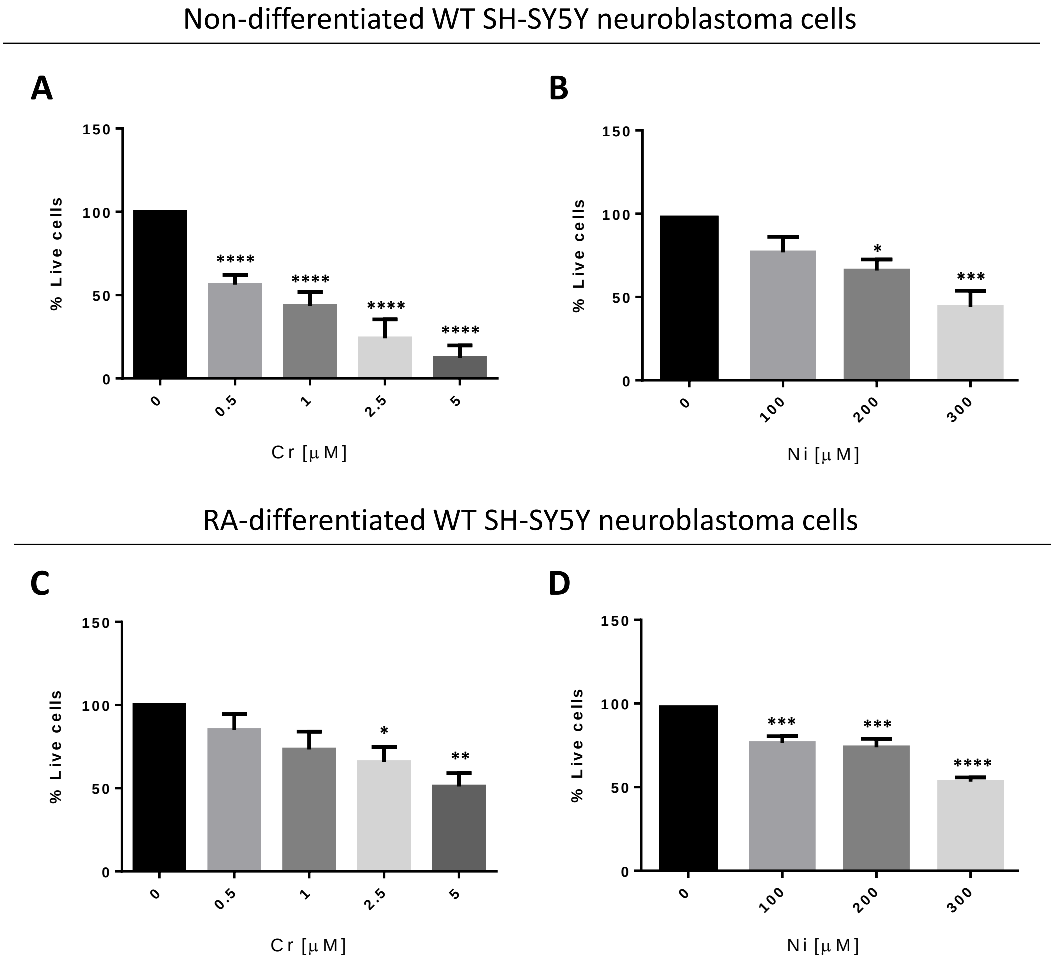

Supplementary Figure 5. Statistical analysis of the MTT assay after Cr and Ni treatment in non-differentiated and RA-differentiated SH-SY5Y neuroblastoma cells.

Plots showing the average ± SEM of the percentage of live cells after treatments with increasing doses of Cr (0-5 μM) and Ni (0-300μM) from 5 independent experiments. The statistical significance was determined by the one-way analysis of variance (ANOVA) using GraphPad Prism 6. (*p<0.5, **p<0.01, ***p<0.001 and ****p<0.0001 heavy metal treated cells significantly different from untreated cells).

Supplementary Figure 6

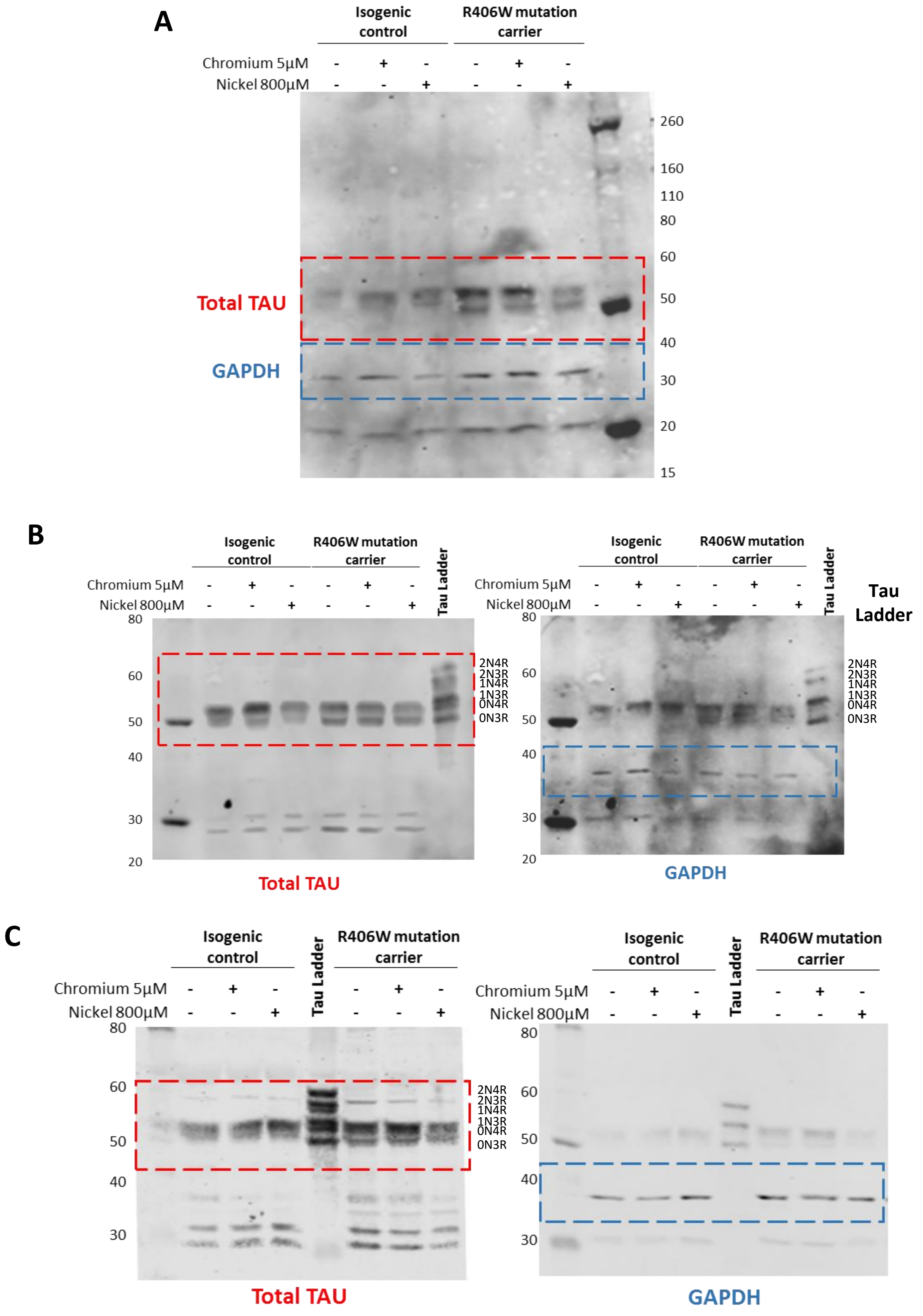

Supplementary Figure 6. Tau and GAPDH immunoblots in iNeurons.
Images of the three independent western blots performed in control and R406W tau mutant iNeurons to detect tau levels (Red Box) before and after the treatment with Cr 5μM and Ni 800μM for 72 hours. GAPDH protein was used as a loading control (Blue box). A) Representative WB used in main figure. B and C) Western blots used for quantification.

Supplementary Figure 7

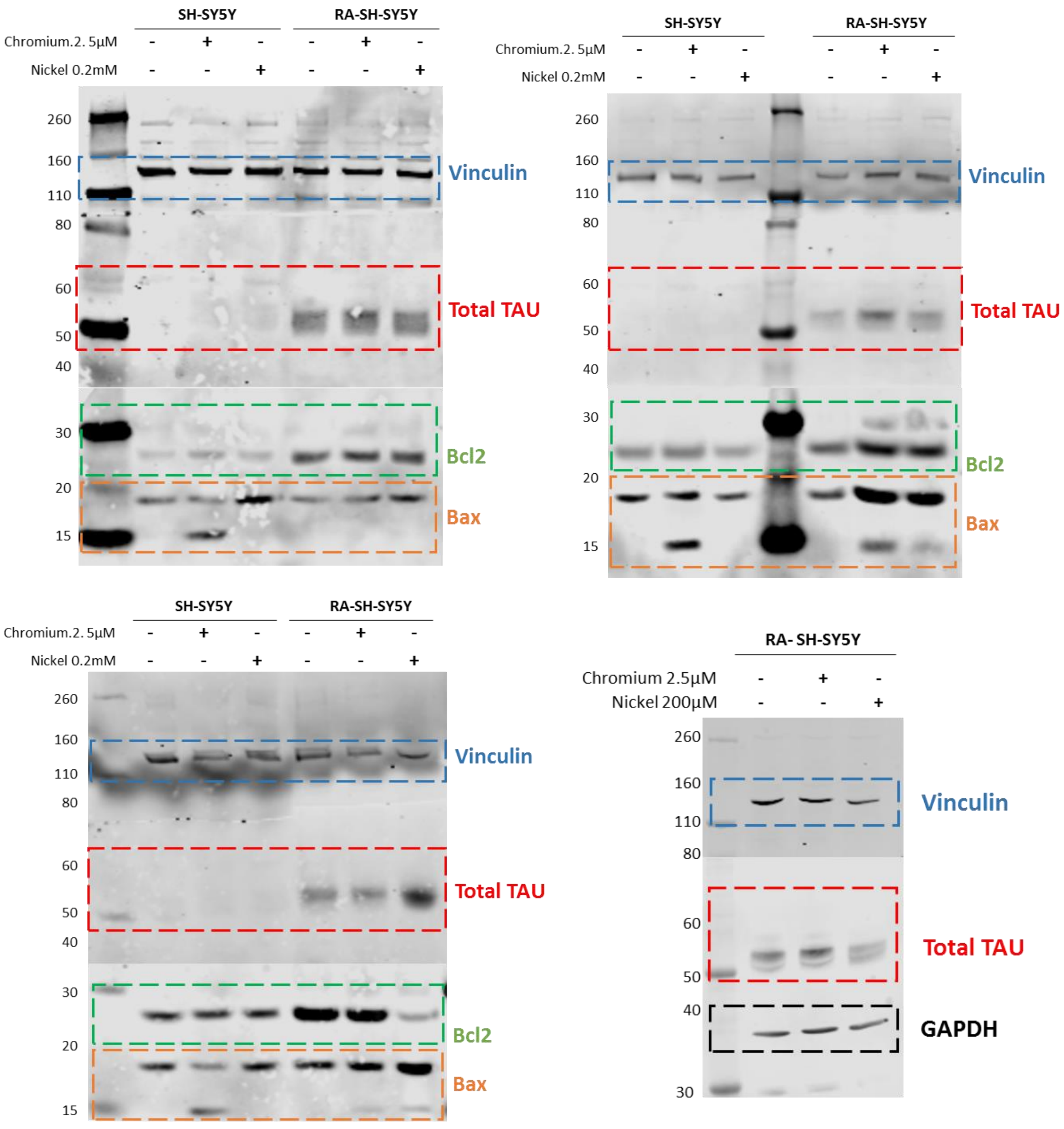

Supplementary Figure 7. Immunoblots in non differentiated and RA-differentiated SH-SY5Y cells. Representative images of all the independent western blots used to detect tau (Red Box), Bcl2 (Green Box), Bax (Orange Box) levels in non differentiated and RA-differentiated SH-SY5Y cells after treatment with Cr 2.5µM and Ni 200µM for 24 hours. Vinculin protein was used as a loading control (Blue Box).

Supplementary Figure 8

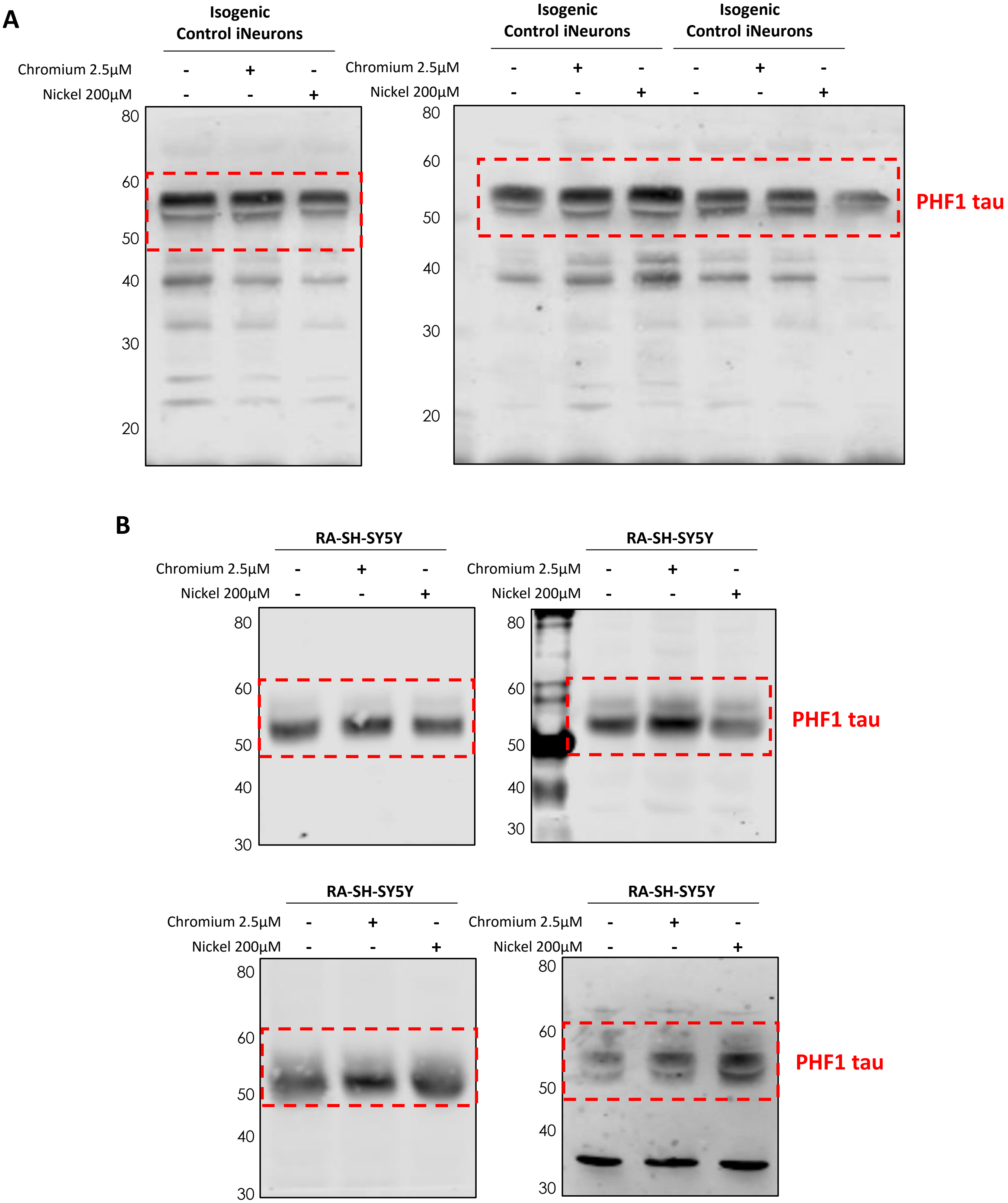

Supplementary Figure 8. PHF1 tau Western blots in iPSC-iNeurons and RA-SHSY5Y cells
Images of the western blots performed in control iNeurons **(A)** and RA-SH-SY5Y cells **(B)** to detect PHF1 (phosphorylated) tau protein (Red Box) before and after chromium and nickel treatments..

Supplementary Figure 9

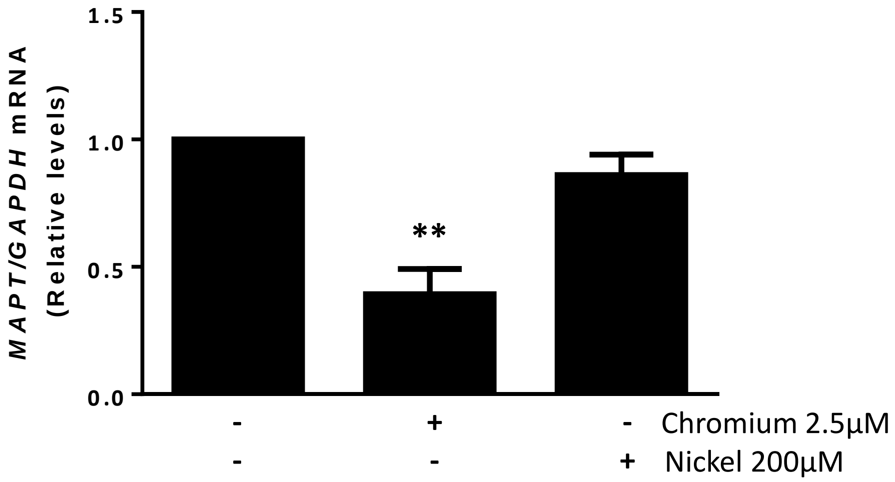

Supplementary Figure 9. Effect of chromium and nickel exposure in *MAPT*/tau mRNA levels.

RA-differentiated SH-SY5Y cells were treated with Cr 2.5µM and Ni 200µM for 24 hours. The levels of tau (*MAPT*) mRNA were measured by quantitative RT-PCR. *GAPDH* was used as housekeeping gene. Plot is showing the average ± SEM of 3 individual experiments. The statistical significance was determined by the one-way analysis of variance (ANOVA) using GraphPad Prism 6. (**p<0.01 statistical significantly different from untreated cells).

Supplementary Figure 10

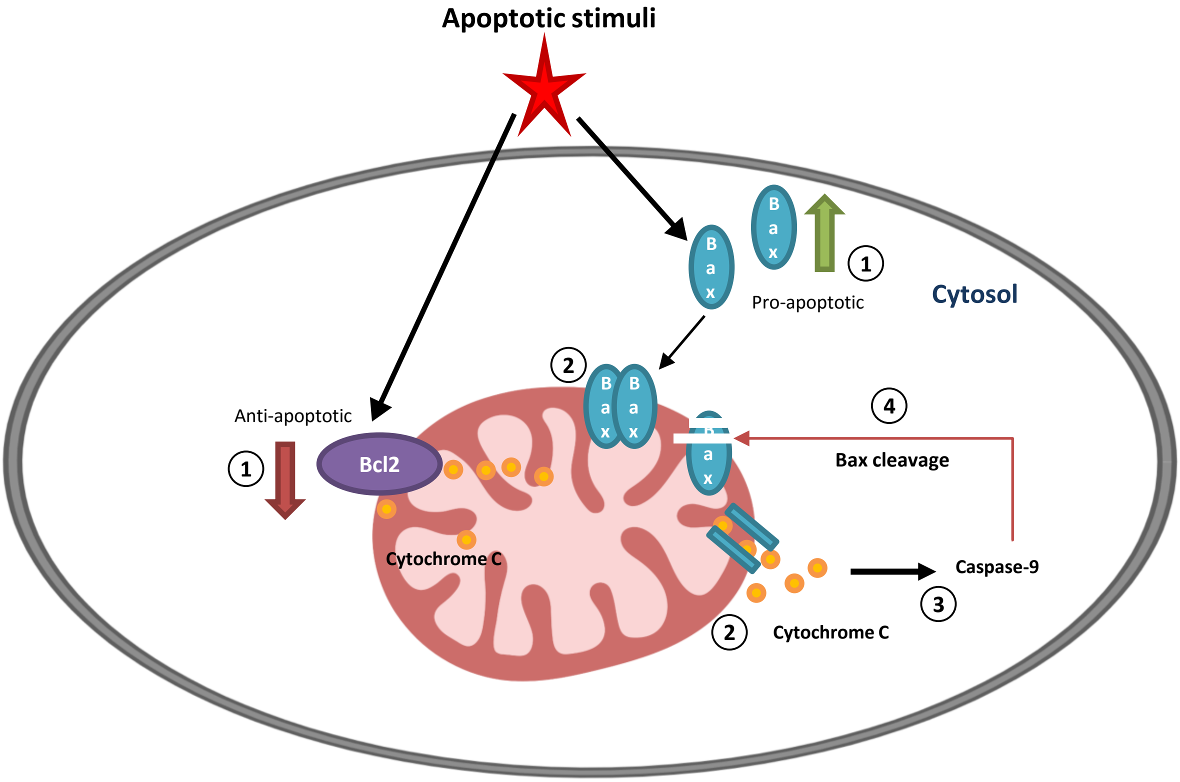

Supplementary Figure 10. Schematic representation of the mitochondrial apoptosis pathway. When the cell is affected by an apoptotic stimuli, the activation of the apoptosis pathway leads to the decrease in levels of anti-apoptotic proteins such as Bcl2 and the increase of pro-apoptotic proteins such as Bax (1). The Bax protein changes its localization from the cytosol to the mitochondria where open pores in the membrane to release cytochrome c (2) (Early apoptosis). (3) In the cytosol, cytochrome c activates apoptosis-related protein such as caspase-9 that, among other functions, induces the cleavage of Bax protein resulting in a 18KD fragment which is more active than full length protein in disrupting the mitochondrial membrane (Late apoptosis) (4).

Supplementary Figure 11

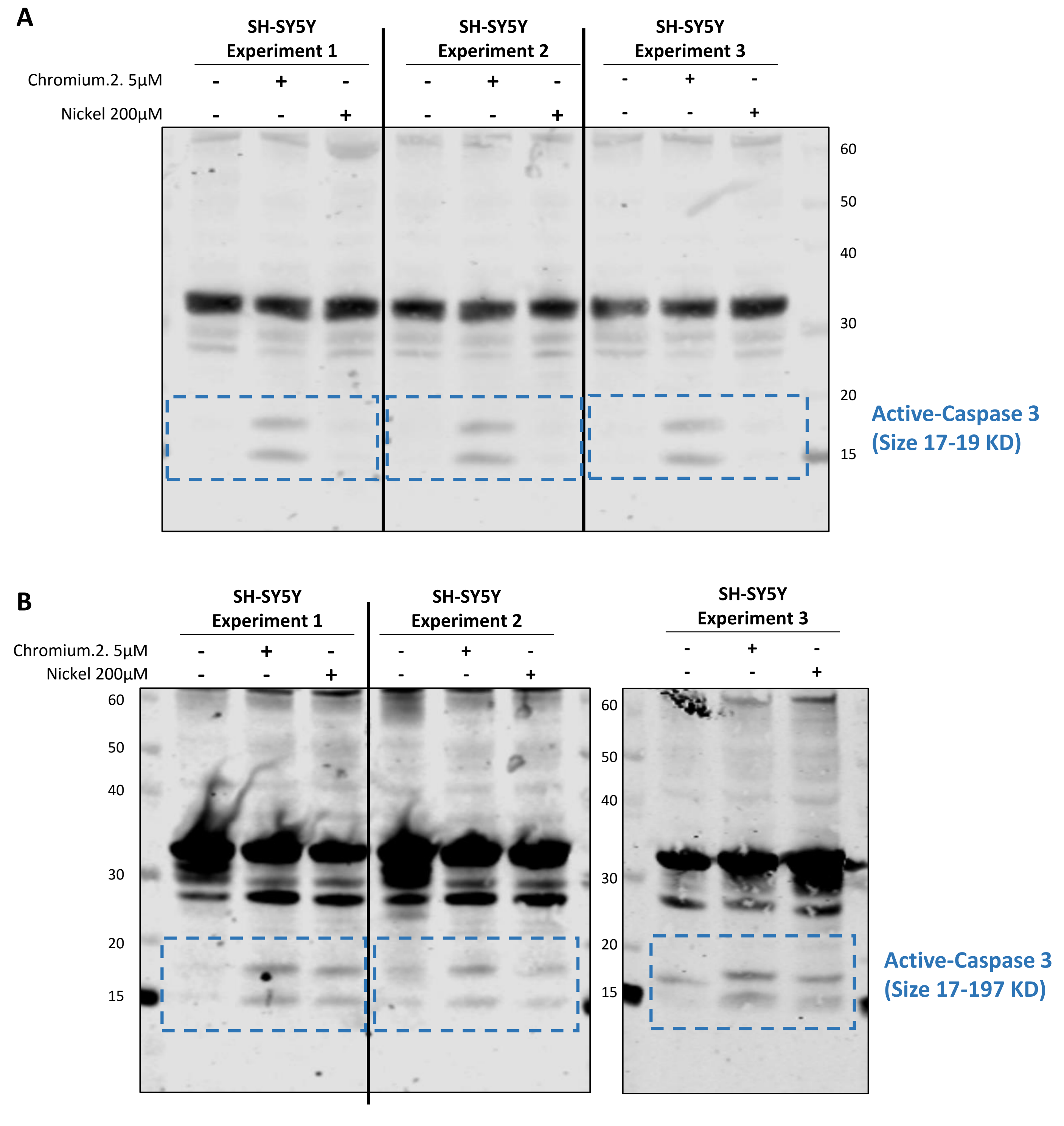

Supplementary Figure 11. Caspase-3 inmmunoblots in non-differentiated and RA-differentiated SH-SY5Y cells.

Image of the membranes used to measure caspase-3 cleavage after Cr and Ni treatment in non-differentiated **(A)** and RA-differentiated SH-SY5Y **(B)** cells. Pro-caspase 3 has a predicted size of 35KD. When apoptosis is activated, pro-caspase 3 is cleaved into small fragments (17-19 KD) of active caspase-3 protein (Blue box).

Supplementary Figure 12

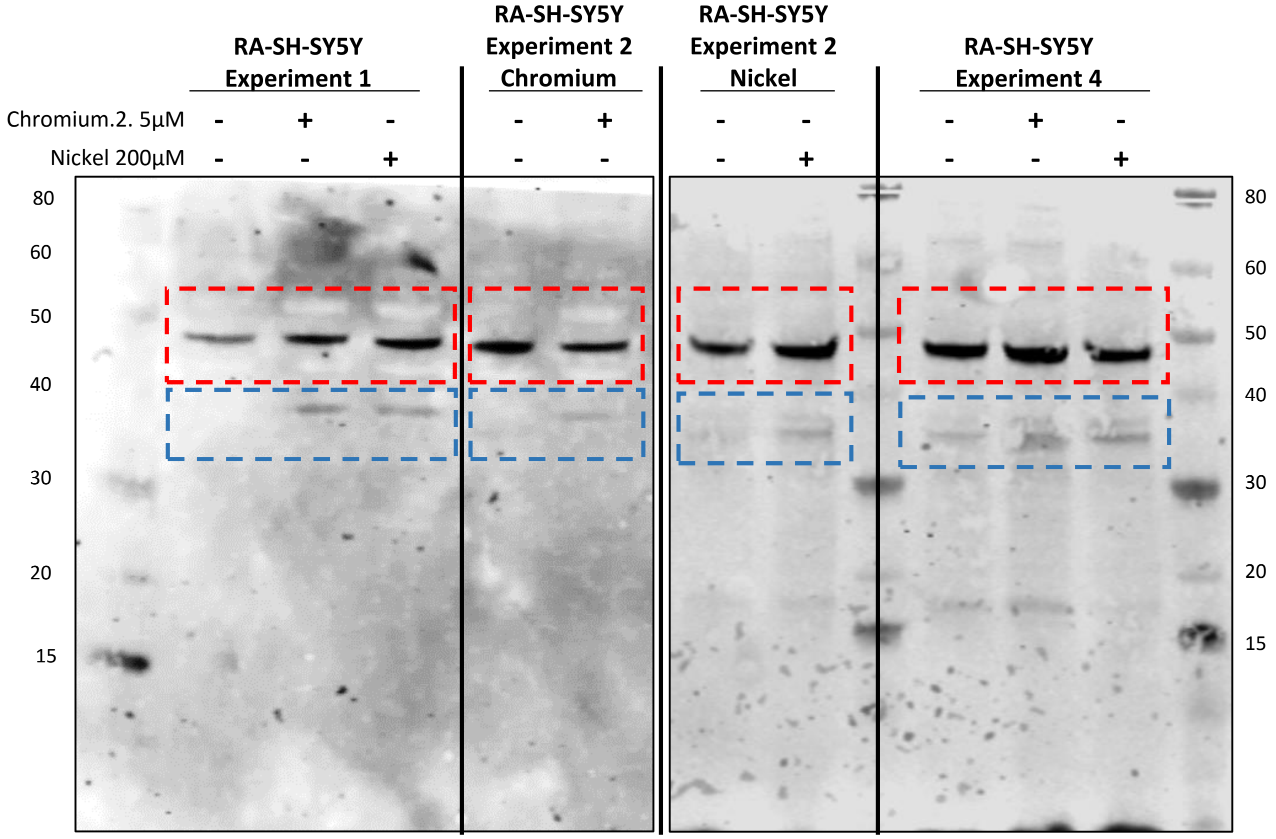

Red Box: Pro-Caspase 9 (Predicted size 46KD)

Blue Box: Active-Caspase 9 (Predicted size 37KD)

Supplementary Figure 12. Caspase-9 inmmunoblots in RA-differentiated SH-SY5Y cells
Image of the western blots used to analyze caspase-9 cleavage after Cr and Ni treatment in RA-differentiated SH-SY5Y cells. Pro-caspase 9 (Red box) has a predicted size of 46KD, the activation of intrinsic/mitochondrial leads to pro-caspase 9 cleavage by cytochrome c into small fragments of 37KD fragments (Blue box). Our results show that Cr and Ni exposure activates caspase-9 in RA-differentiated SH-SY5Y cells.
